# Simple and practical methods for utilizing parylene C film based on vertical deposition and laser patterning

**DOI:** 10.1038/s41598-022-13080-w

**Published:** 2022-06-09

**Authors:** Jee Hoon Sim, Hyeonwook Chae, Su-Bon Kim, Seunghyup Yoo

**Affiliations:** School of Electrical Engineering, Korea Advanced Institute of Science and Technology (KAIST), 291 Daehak-Ro, Yuseong-Gu, Daejeon, 34141 Republic of Korea

**Keywords:** Materials science, Materials for devices, Electronic devices

## Abstract

We propose two novel methods to effectively utilize parylene C films. First, we demonstrate a vertical deposition method capable of depositing a parylene C film of the same thickness on both sides of a sample. Through this method, we have formed parylene C films with a thickness of 4 μm on both sides of the sample with a thickness deviation of less than 2.5%. Further optical verification indicates that parylene C films formed by this method have a very uniform thickness distribution on each side of the surfaces. Second, we propose a debris-tolerant laser patterning method as a mask-less means to fabricate self-supporting ultrathin parylene C films. This method does not involve any photolithography and entails a simple and rapid process that can be performed using only a few materials with excellent biocompatibility. It is demonstrated that patterned parylene C films exhibit a high degree of surface uniformity and have various geometrical shapes so that they can be used for substrates of highly flexible and/or stretchable devices. Finally, we use both of the proposed methods to fabricate flexible, stretchable, and waterproof-packaged bifacial blue LED modules to illustrate their potential in emerging applications that would benefit from such versatile form factors.

## Introduction

**Figure 1:**
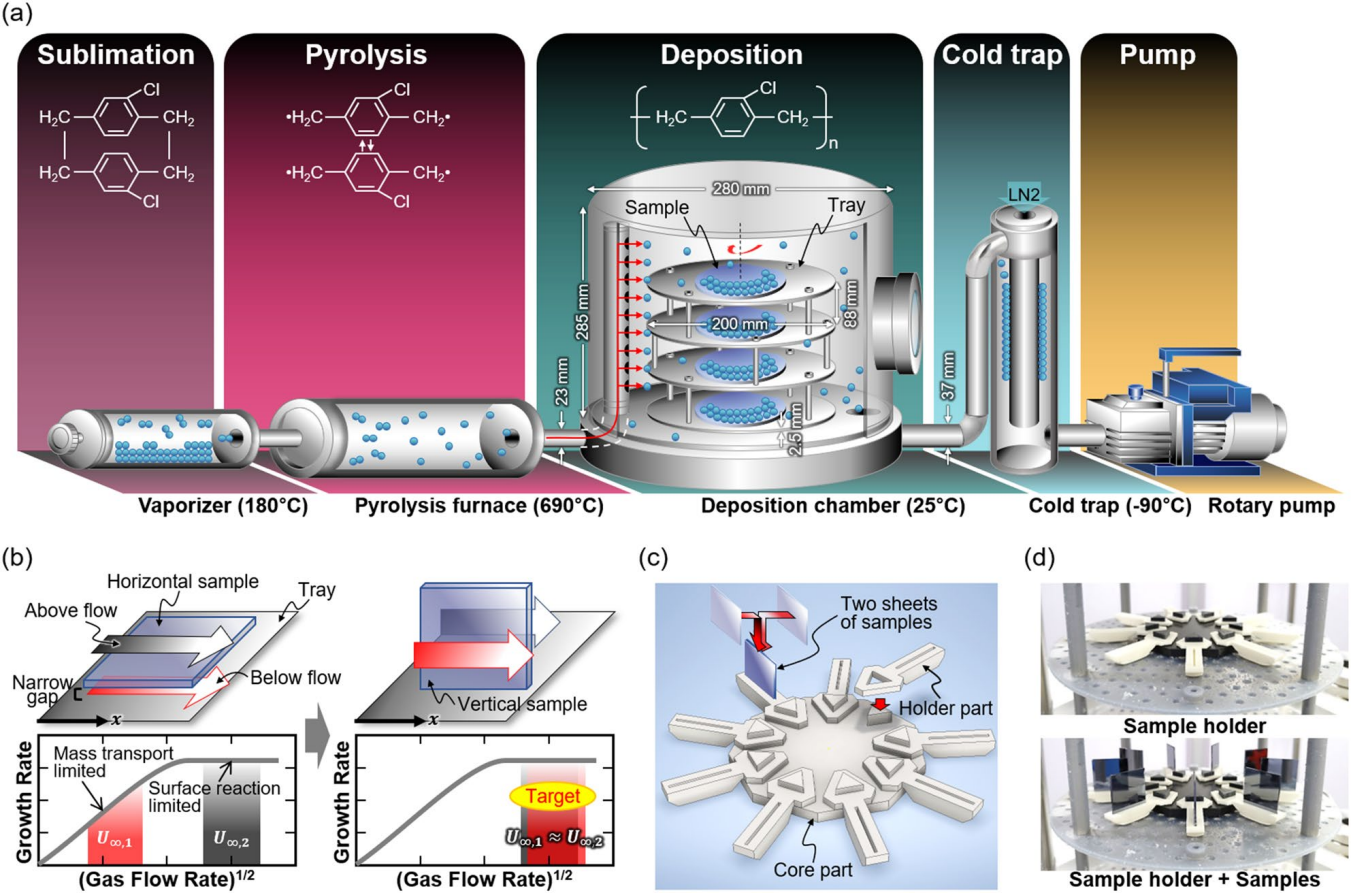
Schematic diagrams of (**a**) the conventional deposition setup for parylene C films and (**b**) the expected parylene C monomer vapor flow in the sample with horizontal and vertical deposition. (**c**) Sample holder layout designed for vertical deposition and (**d**) actual photos of parylene C coating system equipment.

Parylene C films are widely used in various fields due to its excellent optical transmittance^1,2^, waterproofness^3–5^, insulation^6,7^, and biocompatibility^8–11^. Recently, a wide range of applications ranging from substrates and packaging layers of wearable devices for healthcare^12,13^ to thin film encapsulation layers of flexible OLEDs have been demonstrated^14,15^. In this article, we describe how parylene C film can be used more effectively in these various fields. The basic deposition process of parylene C film^16,17^ is schematically illustrated in Fig. 1a. First, parylene C powder in the form of a dimer is sublimated in a vaporizer at 180 °C, and then the parylene C vapor passes through a furnace at 690 °C and becomes a monomer through pyrolysis. Next, when the monomer with radicals enters the deposition chamber at room temperature and touches the sample surface and polymerizes, a parylene C film is formed. At this time, the sample tray rotates continuously to ensure uniform film thickness. The behavior of the monomer vapor flow in the deposition chamber is simulated by COMSOL Multiphysics software, as shown in Supplementary Fig. S1. A cold trap containing liquid nitrogen is situated between the chamber and the rotary pump, which induces polymerization of residual monomer vapor to prevent damage to the rotary pump.

In this article, we report on two novel methods that can utilize parylene C film more effectively. First, we propose a vertical deposition method as a single step bifacial deposition method that can solve the problem of thickness difference on both sides of the sample that occurs during conventional horizontal deposition^18^. We thus intend to overcome the limitations of horizontal deposition by identifying the principle of the vertical deposition method and proving its effectiveness through measurement and analysis. Second, we propose a debris-tolerant laser patterning method, which is presented as a simpler and more biocompatible process than the conventional parylene C patterning process using photoresist chemicals^19,20^. The effectiveness of each of the proposed methods is illustrated by fabricating and demonstrating flexible, stretchable, and waterproof-packaged bifacial blue LED modules to which both of the aforementioned methods are applied.

## Experiments and results

### Vertical deposition method

In general, when parylene C deposition is carried out with the sample in a horizontal direction, films are formed on both the upper side and lower side surfaces of the sample, but they do not have the same thickness. The explanation for this is delineated by Eq. (1) below representing the thickness of the boundary layer^21,22^.

1 $$ \delta \left( x \right) \sim \sqrt {\frac{\mu x}{{\rho U_{\infty } }}} $$

where $\mu$, $\rho$, and $U_{\infty }$ denote the viscosity, density, and flow rate of the monomer vapor, respectively, and $x$ is the distance the vapor moved from the sample boundary. In the case of vapor flow above and below the substrate in horizontal deposition scheme shown in the left of Fig. 1b, $\mu$, $\rho$, and $x$ in Eq. (1) are all the same. However, in the case of the flow rate of the monomer vapor below the substrate ($U_{\infty ,1}$) has a relatively small value due to the increase in friction caused by the narrow gap between the sample and the tray. In this case, when the flow rate is slow, the thickness of the boundary layer is large and the growth rate has a mass transport limited state^23^. Conversely, in the case of the flow above the substrate, the mass transport rate becomes faster than the surface reaction rate since the flow rate ($U_{\infty ,2}$) is relatively high and the thickness of the boundary layer is sufficiently small, and the growth rate consequently becomes constant regardless of the flow rate^24^. In short, since the up-side flow has a higher growth rate than the down-side flow, the final thickness is also thicker for the films on the upper side than for those on the lower side. In this article, we thus propose a vertical deposition method, as shown on the right side of Fig. 1b, to overcome the thickness imbalance resulting from a conventional, horizontal deposition method. If the rates of both flows are equal by exposing both surfaces of a sample in a symmetrical configuration through the proposed vertical deposition, the boundary layer thicknesses will be equal. As a result, the growth rate also becomes the same, and uniform thickness of the parylene C films may be achievable for both sides of the sample.

We designed the sample holder so that it can be easily applied to the parylene C coating system equipment generally used in the laboratory, and the layout is shown in Fig. 1c. The sample holder consists of one core part and nine holder parts that can be selected according to the type and size of the sample, all of which were printed using a 3D printer (Single Plus-310F of Cubicon, Rep. of Korea). We used a silicon wafer cut in a square shape with a side length of 1-inch as a sample, and stacked two sheets so that all deposition surfaces were the smooth surface of the wafer. Figure 1d is a picture of the sample holder placed on the sample tray, and a video showing the actual rotation can be seen in Supplementary Video S1.

**Figure 2:**
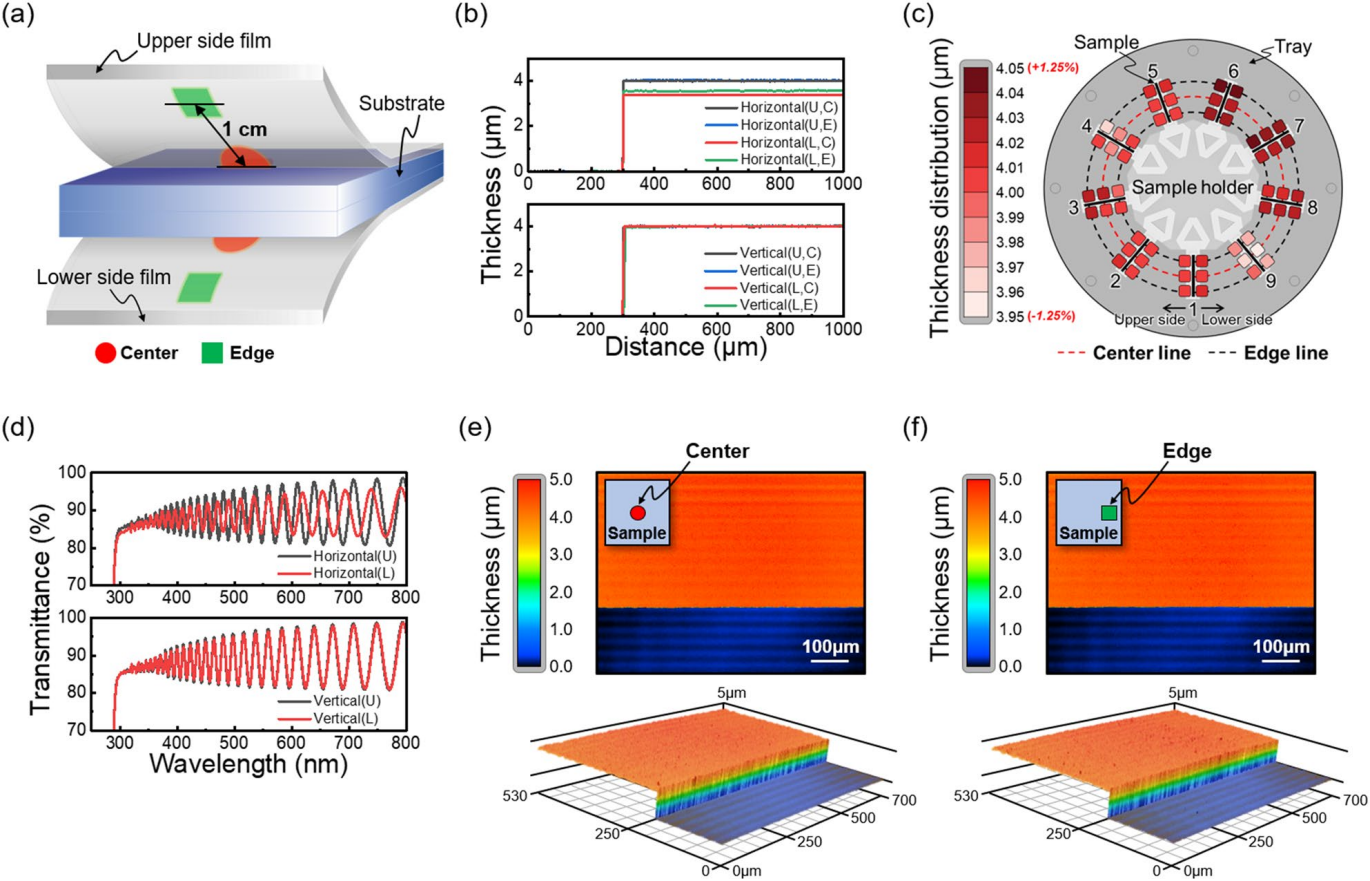
(**a**) A schematic diagram of the two-sided deposition sample of parylene C film. (**b**) Film thickness measurement results in all cases, and (**c**) thickness distribution of vertical deposition samples. (**d**) Transmittance measurement results in all cases, and surface morphology measurement results for (**e**) center and (**f**) edge area of vertical deposition samples.

Prior to the measurement, we specified the measurement location on the film formed on the sample. A schematic diagram divided by a red circle marked at the center of the sample and a green square at the edge 1 cm away from the center is shown in Fig. 2a. We sought to form a parylene C film with a thickness of 4 μm, and after placing the samples in the equipment (product of Young Hi-Tech, Rep. of Korea) in the horizontal and vertical directions, respectively, deposition was carried out. O_2_ plasma etching was then performed in advance to clearly distinguish the substrate and film boundary, and the recipe and process are summarized in Supplementary Fig. S2.

The parylene C film thickness was measured using a stylus profilometer (P-15 of KLA-Tencor), and the results are shown in Fig. 2b. U, L, C, and E are abbreviations for Upper side, Lower side, Center, and Edge, respectively. In the case of horizontal deposition, the thickness of the lower side film is thinner than that of upper side film, which is ascribed to the difference in the growth rate according to the reduced flow rate, as mentioned above. To make matters worse, the thickness at the central part of the lower side film turns out to be thinner than the edge part, which can be regarded to come from the micro gap formed between the substrate and the tray^18^. In the case of vertical deposition, on the other hand, the thicknesses of both the upper side and lower side films converge to 4 μm, because a sufficient flow rate acts on both surfaces and the boundary layer thickness becomes sufficiently small, resulting in a surface reaction limited state. To assess the thickness uniformity of each of the samples used in the vertical deposition, we checked the thickness distribution at the center and the edge in the upper side and lower side films for a total of nine samples. As shown in Fig. 2c, we were able to confirm that parylene C films made with the vertical deposition exhibit a very uniform thickness distribution within an error range of 2.5% with respect to the target thickness of 4 μm.

Transmittance spectra of the films measured using a UV–Vis spectrometer (LAMBDA 950 of PerkinElmer) also show that their sinusoidal modulation due to the interference effect coincides well between the upper-side and the lower-side films in the case of vertical deposition unlike the case of horizontal deposition. (Fig. 2d). Because the modulation period and the overall transmittance value are both dependent on the thickness of the films, the observed results are consistent with the measurement done with the surface profiler. Furthermore, the overall transmittance is higher than 80% in the entire visible light region, being consistent with the inherent high transparency of the parylene C film^25^. Finally, to verify the uniformity of the parylene C film once more, the surface morphology of the center and the edge of the vertically deposited sample was checked through a 3D laser scanning microscope (VK-X200 series of KEYENCE), and the results are shown in Fig. 2e, f. The result clearly shows that both the center and the edge part show a very uniform film distribution, which demonstrates the effectiveness of the proposed vertical deposition method. As such, we have demonstrated that a parylene C film of the same thickness with a uniform distribution on both sides of the sample can be formed through the vertical deposition method. Using this method, only one deposition step is required to form a parylene C film of the same thickness on both sides of the sample, and thus the number of processes and dimer consumption can both be reduced by half compared to the conventional horizontal deposition method. For example, it will be advantageous in applications such as flexible OLEDs having multiple layers of parylene C film as an encapsulation film.

### Laser patterning method

Recently, the use of parylene C film as a substrate for wearable devices for healthcare is increasing due to the unique biocompatibility, flexibility, and high transparency of the material. Parylene C substrates have various shapes and structures according to their respective functions and specifications, and O_2_ plasma etching has been popularly used as a film patterning method^19,20^. However, in the case of this method, since a photoresist chemical is generally used, in terms of biocompatibility, it may be unsuitable for use as a wearable device for healthcare. In addition, the process may be complicated because it is necessary to create and remove a photoresist or metal film serving as an etching mask. Therefore, we propose a method for manufacturing a parylene C substrate through laser patterning that is not only harmless to the human body, but also easy to design and change, and the overall process time can be made very short.

**Figure 3:**
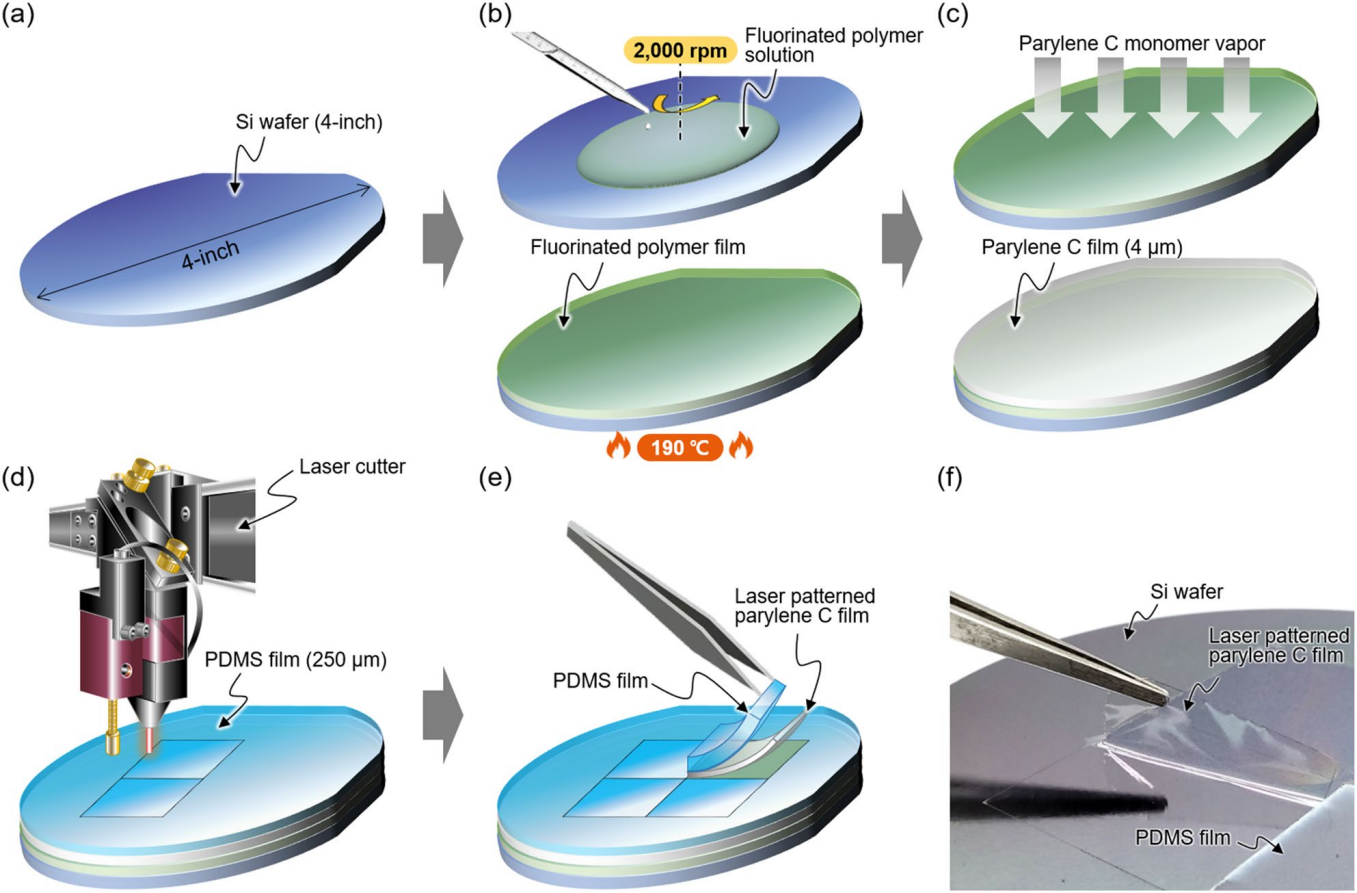
Parylene C substrate manufacturing process through laser patterning: (**a**) 4-inch bare silicon wafer, (**b**) spin coating of fluorinated polymer, (**c**) 4 μm thick parylerne C film deposition, (**d**) substrate patterning using laser cutter, and (**e**) peeling off the parylene C substrate. (**f**) Actual photo of the produced parylene C substrate.

The process started with a 4-inch diameter silicon wafer as shown in Fig. 3a. Next, as shown in Fig. 3b, 2.5 ml of fluorinated polymer (Novec™ 1700 Electronic Grade Coating of 3M™) was first spin-coated at 2000 rpm for 30 s before parylene C film deposition, and then annealed on a hot plate at 190 °C for 10 min. This fluorinated polymer facilitates removal of the parylene C film from the silicon wafer, and is a safe material that is not classified as a hazardous material by OSHA Hazard Communication Standard, 29 CFR 1910.1200. It should be noted that handling should be done with caution as liquid fluorinated polymers harden immediately upon exposure to atmospheric conditions. A 4 μm-thick parylene C film was subsequently deposited, as shown in Fig. 3c. Next, as shown in Fig. 3d, after covering the parylene C film with the PDMS film (HT6240 of Rogers, 250 μm-thick), patterning was carried out according to the designed layout using a laser cutter (BG-GTC 3050N of BUGWANG GTC, Rep. of Korea). Detailed specifications of the laser cutter are summarized in the Supplementary Fig. S3. In this scheme, the PDMS film played a key role in preventing small particles generated during laser patterning from being adsorbed onto the parylene C film. (Refer to Supplementary Fig. S4 for the SEM images (UFR FEG-SEM of FEI company) showing the difference in particle distribution on the surface of parylene C films according to the use of the PDMS film during laser patterning) Finally, as shown in Fig. 3e, the PDMS film and the parylene C film can be easily peeled off sequentially using tweezers, and an actual photograph is shown in Fig. 3f. In this introduction to the process, we employed a simple rectangular parylene C substrate as an example, and more diverse and complex patterns will be described later.

**Figure 4:**
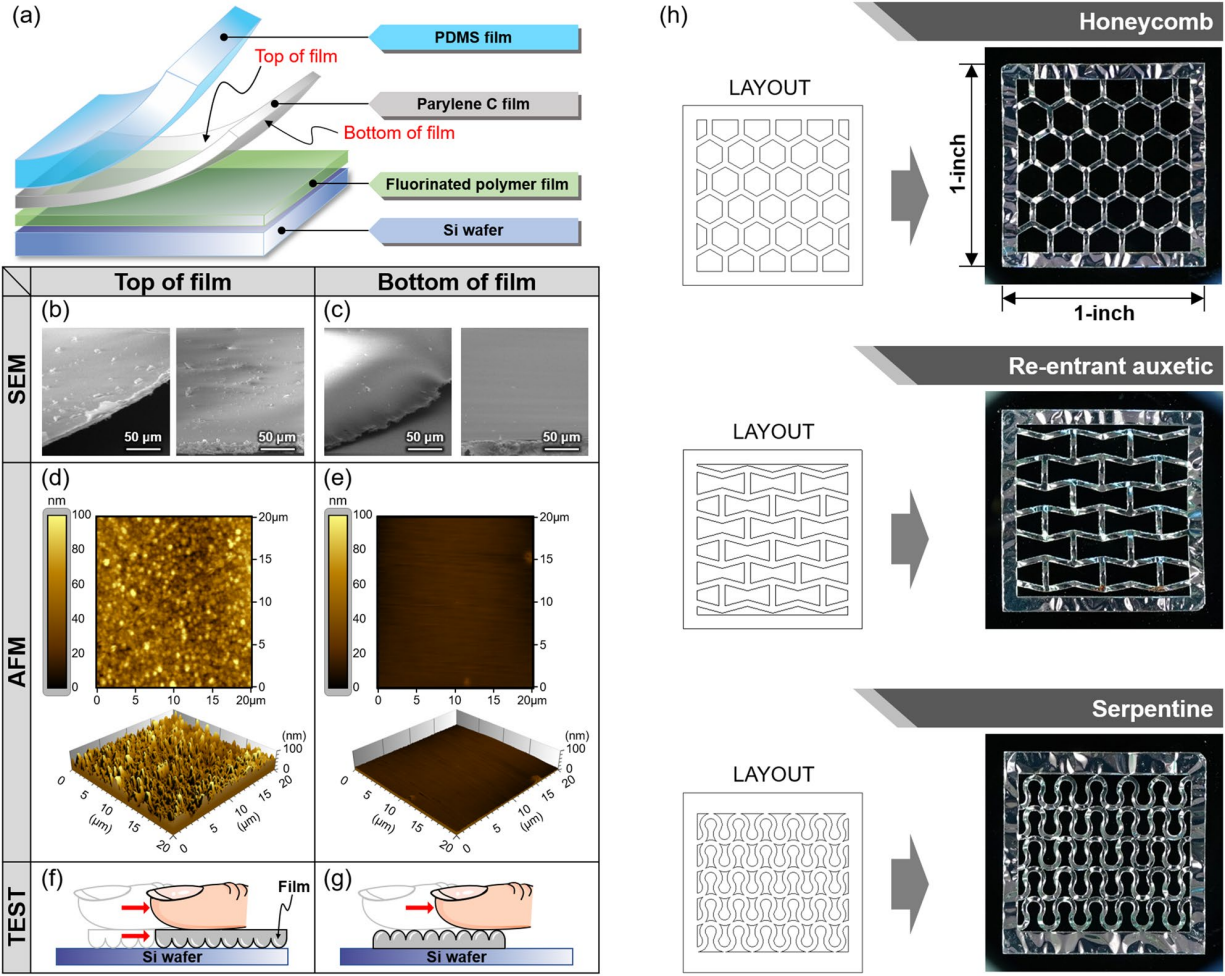
(**a**) The layer structure of an assembly used for laser patterning of parylene C films. SEM images of the boundary surface at (**b**) top and (**c**) bottom of the film. Roughness images of (**d**) top and (**e**) bottom of film using AFM. Schematic diagrams of sliding test to compare the roughness between (**f**) top and (**g**) bottom of the film. (**h**) Examples demonstrating various patterns with 4 μm parylene C film using laser patterning: honeycomb, re-entrant auxetic, and serpentine structures.

We next looked more closely at the surface of the parylene C substrate fabricated by the laser patterning method. As shown in Fig. 4a, the area where the parylene C film was in contact with the PDMS film was defined as the top of film, and the area in contact with the fluorinated polymer film was defined as the bottom of film. First, in the case of the top of the film, it was possible to confirm through the SEM image in Fig. 4b that many particles were present at the cutting boundary despite the blocking effect of the PDMS film. On the other hand, in the case of the bottom of the film, it could be confirmed from Fig. 4c that the amount of particles adsorbed was relatively small as the parylene C film was attached to the fluorinated polymer film. A more pronounced difference between the two surfaces was seen through an AFM analysis (NaniteAFM & C3000 of Nanosurf). In the case of the top of the film, a hillocked morphology^26^, a typical surface feature of parylene C film, was revealed as shown in Fig. 4d, and the RMS roughness value was 16.8 nm. On the other hand, in the case of the bottom of the film, it could be confirmed from Fig. 4e that it was directly affected by the silicon wafer surface coated with fluorinated polymer film and had a very smooth surface, and the RMS roughness value was as low as 2.87 nm. The RMS roughness value of the silicon wafer surface coated with fluorinated polymer film was 2.05 nm, as confirmed by the AFM measurement result for this surface shown in Supplementary Fig. S5. Furthermore, it was confirmed that the thermal effect generated by laser cutting had no significant effect (bubbles or cracks^27^) on the surface roughness of parylene C film at cutting boundary, which is shown in the Supplementary Fig. S6.

For direct verification of the difference in roughness, a test was conducted to move the parylene C film on a silicon wafer with a finger, and schematic diagrams are shown in Fig. 4f, g. As expected, the top of the film with high roughness slid as well because the contact area with the silicon wafer was narrow, and the bottom of the film with low roughness did not slide as well because the contact area was wide. The actual test video can be seen in Supplementary Videos S2 and S3. In conclusion, we recommend using the bottom of film with low particle contamination at the cutting boundary and excellent surface roughness as a substrate. This is because the characteristics and stability of the electrical components on the surface are further guaranteed when being formed on a smooth surface.

As a next step, we demonstrated parylene C substrates with various patterns. Each unit pattern was designed to be an array in a square lattice with a side of 1-inch in length, and the layouts and actual photos in the Fig. 4h. We designed a total of three patterns ranging from honeycomb^28,29^, re-entrant auxetic^30–33^, and serpentine^34–37^ structures. We confirmed that all the patterns of the parylene C substrate were formed delicately at the same level as the layout. This laser patterning method, which can freely produce patterns of any shape, such as curved surfaces and straight lines with a width of several hundred micrometers, will contribute to the simple and fast production of ultrathin parylene C films that can be used as substrates in various applications.

### Demonstration of bifacial blue LED modules

**Figure 5:**
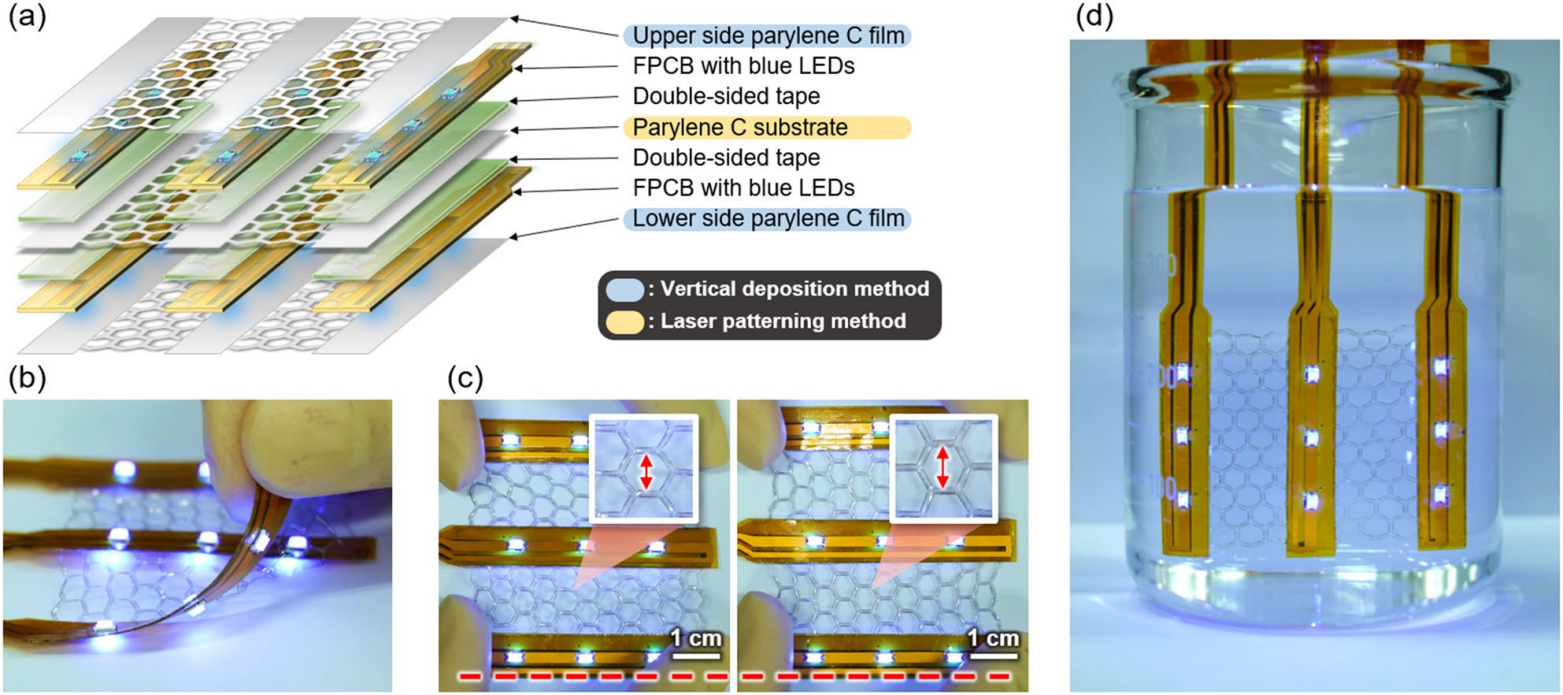
(**a**) Schematic diagram and actual photos of (**b**) flexibility, (**c**) stretchability, and (**d**) waterproof test of blue LED device.

Finally, we tried to demonstrate the effectiveness of the two process methods proposed in this article through fabrication of exemplary bifacial devices that are flexible, stretchable, and water-proof. (See Fig. 5a for the schematic diagram.) First, the laser patterning method was used to prepare a 4 μm-thick parylene C substrate for which a honeycomb structure was applied among three rigid islands. Next, flexible printed circuit boards (FPCBs) with blue LEDs (CMD15-21UBC/TR8 of VCC) mounted on both sides of the rigid islands were attached with the help of double-sided tapes (467MP of 3M). Additional 4 μm thick parylene C films were subsequently deposited on both sides through the vertical deposition method for passivation of the device. As shown in Fig. 5b and c, it is demonstrated that the completed devices can be bent or stretched to cope with various demand for deformation; for instance, the area of the honeycomb structure was shown to be stretchable by about 15% in one direction without any damage. Furthermore, parylene C passivation layers the whole unit ensured LEDs on both sides to operate even with the unit immersed in water thanks to the water-proof capability and excellent conformability of the parylene C films made with the vertical deposition method. (See Fig. 5d and Supplementary Video S4.).

## Conclusion

In summary, we have introduced and demonstrated novel methods to prepare and utilize parylene C films in a simple yet practical manner. First, we demonstrated a new vertical deposition method that can form a parylene C film with the same and uniform thickness on both sides of the sample to overcome the limitations in the horizontal deposition method. Next, we demonstrated a debris-tolerant method to fabricate high-quality ultrathin parylene C substrates with various shapes and structures through PDMS-assisted laser patterning. Both of the methods were used to prepare electronic devices that can be bent, stretched, and water-proof, illustrating the immense potential and versatility of the proposed methods. We anticipate that the approaches introduced in this work will be highly useful in realizing many emerging electronic devices such as wearable devices for body-attachable applications where both deformability and biocompatibility matter.

## Supplementary Information

Supplementary Legends.

Supplementary Figures.

Supplementary Video S1.

Supplementary Video S2.

Supplementary Video S3.

Supplementary Video S4.

## Data Availability

The datasets generated and analysed during the current study are available from the corresponding author on reasonable request.
