# Supplementary material for: Simple and practical methods for utilizing parylene C film based on vertical deposition and laser patterning
Source: Sci Rep. 2022 Jun 9;12:9506. doi: 10.1038/s41598-022-13080-w (PMC9184507; doi:10.1038/s41598-022-13080-w)
Supplement: Supplementary file 1 — Supplementary Legends. [file 41598_2022_13080_MOESM1_ESM.docx]

Supplementary Information (about video) for

Simple and practical methods for utilizing parylene C film based on vertical deposition and laser patterning

# Jee Hoon Sim1,+, Hyeonwook Chae1,+, Su-Bon Kim1 , and Seunghyup Yoo1,*

1School of Electrical Engineering, Korea Advanced Institute of Science and Technology (KAIST), 291 Daehak-Ro, Yuseong-Gu, Daejeon 34141, Republic of Korea

*[syoo.ee@kaist.edu](mailto:corresponding.author@email.example)

+these authors contributed equally to this work

This file includes:

Heading and corresponding legend of Video S1–S4

**Video S1 (Actual rotation of the sample holder)**: A video shows the actual rotation of the sample holder placed on the sample tray.

**Video S2 (Sliding test of the top side parylene C film)**: A video shows that the top side parylene C film contacted with a bare Si wafer slides well because the contact area is narrow.

**Video S3** **(Sliding test of the bottom side parylene C film)**: A video shows that the bottom side parylene C film contacted with a bare Si wafer does not slide well because the contact area is wide.

**Video S4 (Waterproof test of the bifacial blue LED device)**: A video shows that the bifacial blue LED device emits light without any problem even in the water because of the water-proof capability of the parylene C films.
