# Supplementary material for: Simple and practical methods for utilizing parylene C film based on vertical deposition and laser patterning
Source: Sci Rep. 2022 Jun 9;12:9506. doi: 10.1038/s41598-022-13080-w (PMC9184507; doi:10.1038/s41598-022-13080-w)
Supplement: Supplementary file 2 — Supplementary Figures. [file 41598_2022_13080_MOESM2_ESM.docx]

Supplementary Information for

Simple and practical methods for utilizing parylene C film based on vertical deposition and laser patterning

# Jee Hoon Sim1,+, Hyeonwook Chae1,+, Su-Bon Kim1 , and Seunghyup Yoo1,*

1School of Electrical Engineering, Korea Advanced Institute of Science and Technology (KAIST), 291 Daehak-Ro, Yuseong-Gu, Daejeon 34141, Republic of Korea

*[syoo.ee@kaist.edu](mailto:corresponding.author@email.example)

+these authors contributed equally to this work

This file includes:

Supplementary Figure S1-S6


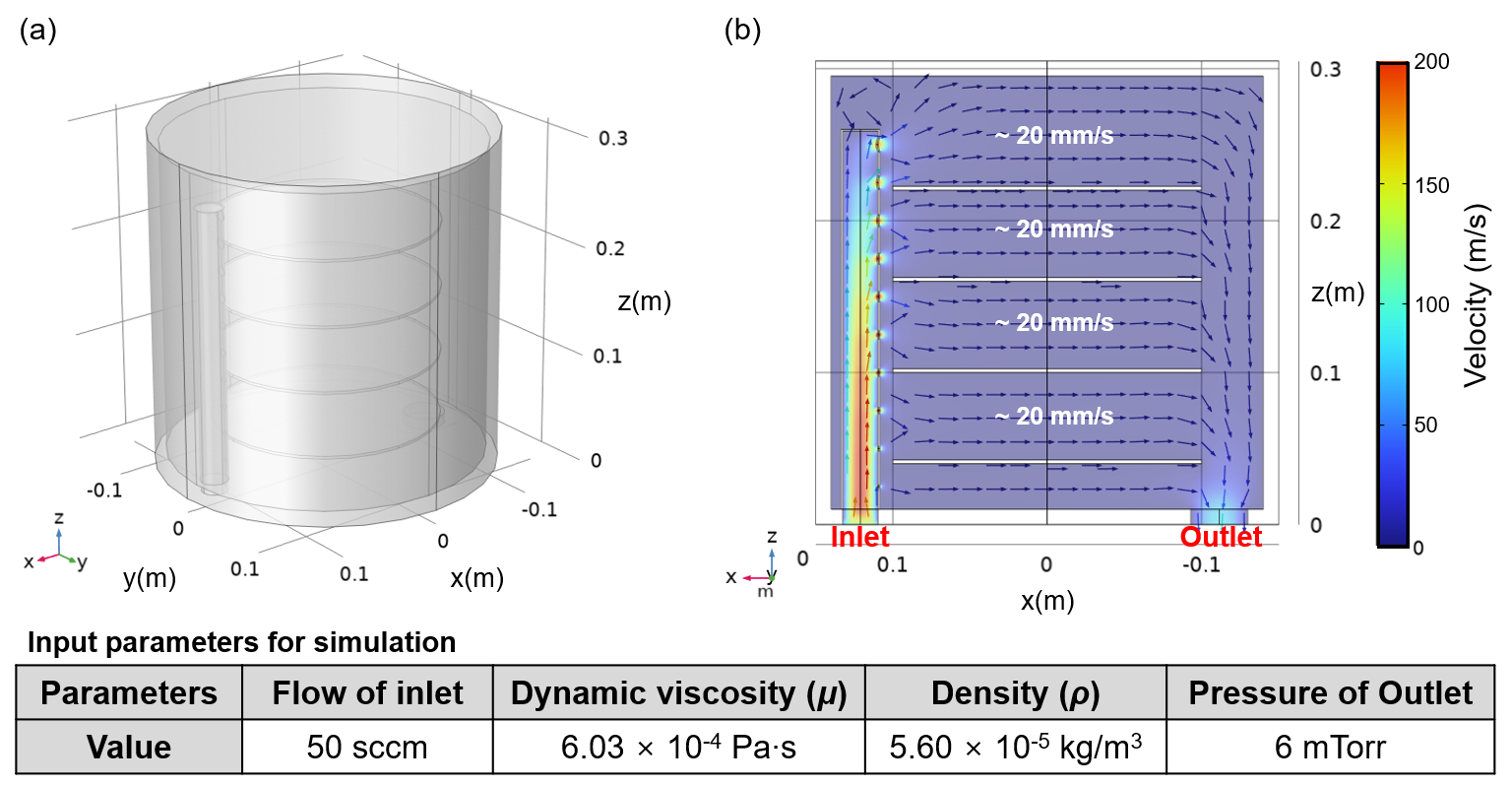


Figure S1. (a) 3D model of deposition chamber for simulating a flow of monomer vapor using COMSOL. (b) A result of finite element analysis to estimate a monomer flow in the inlet/outlet chamber system.

The dynamic viscosity is mentioned in Section 5 of the Ref. 1, and the density was calculated from the general gas equation. Both values were considered at room temperature of 25 ℃.


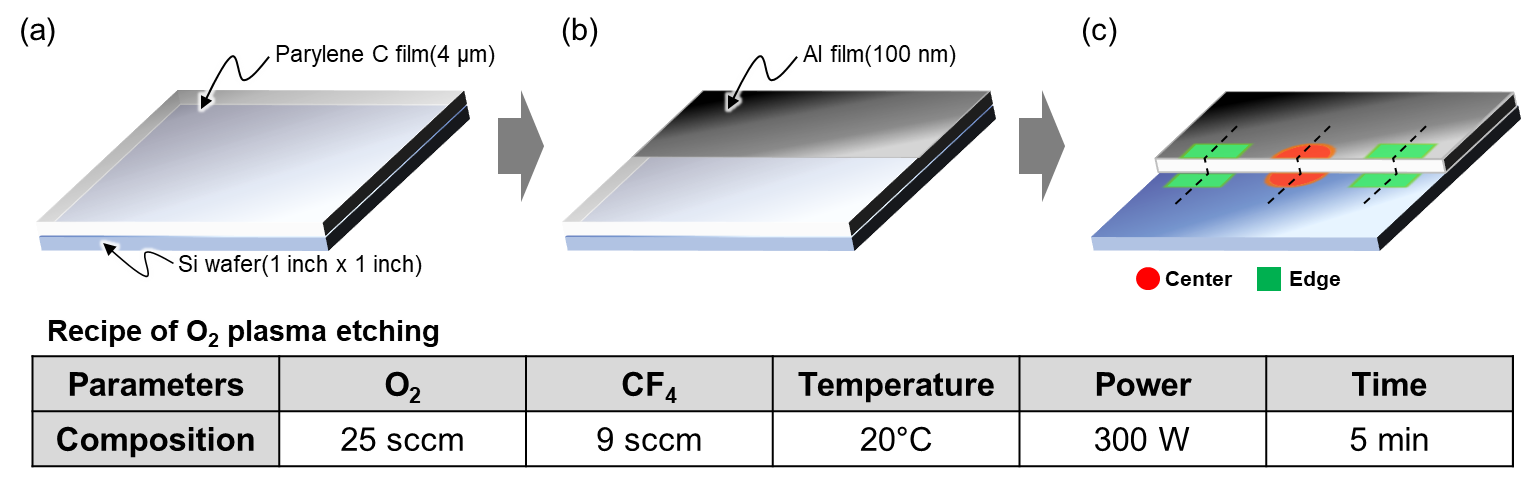
After the parylene C film was deposited as shown in Fig. S2a, 100 nm thick Al was deposited on the half area of the sample as shown in Fig. S2b. At this time, the Al film acted as a mask for the O_2_ plasma etching process. O_2_ plasma etching was performed according to the recipe described in the table, and as shown in Fig. S2c, only the parylene C film was etched where the Al film did not exist. Afterwards, the thickness of the parylene C film was measured for each of the center and edge regions. When measuring with a stylus profilometer, the thickness of the Al film was removed, and when measuring with a 3D laser scanning microscope, it was left as it is. This is because, in the former case, it was the key to identifying the exact thickness of the film, and in the latter case, it was the key to confirming the uniformity of the film.

Figure S2. O_2_ plasma etching process (a-c) and recipe for parylene C film thickness measurement.


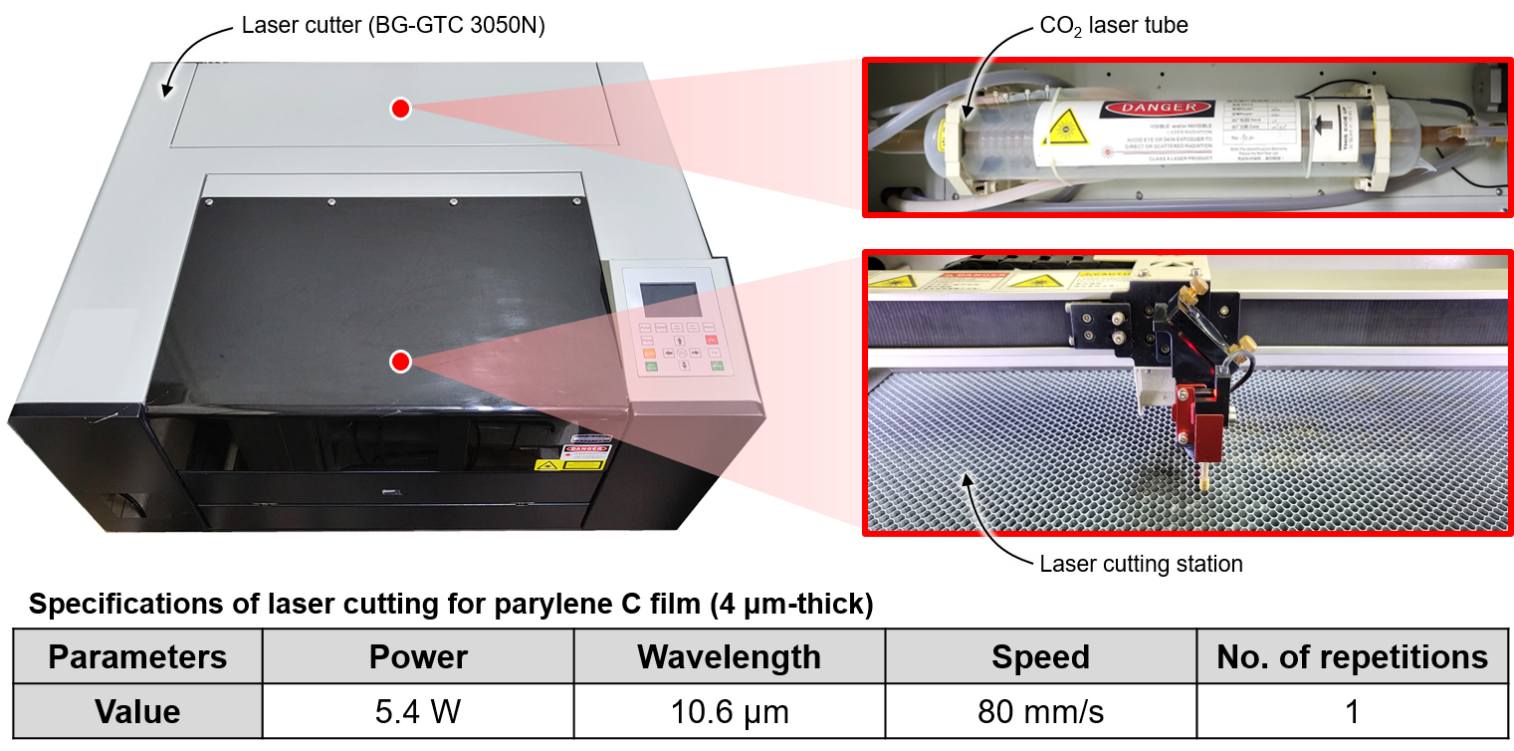
 The maximum power of the CO_2_ laser tube is 45 W, and 5.4 W (12%) was used to cut a 4 μm-thick parylene C film. According to the above specifications, the parylene C film can be cut without any problems even when the commercial PDMS film having a thickness of 250 μm is covered.

Figure S3. Actual photos of laser cutter and specifications of laser cutting for parylene C film (4 μm-thick).


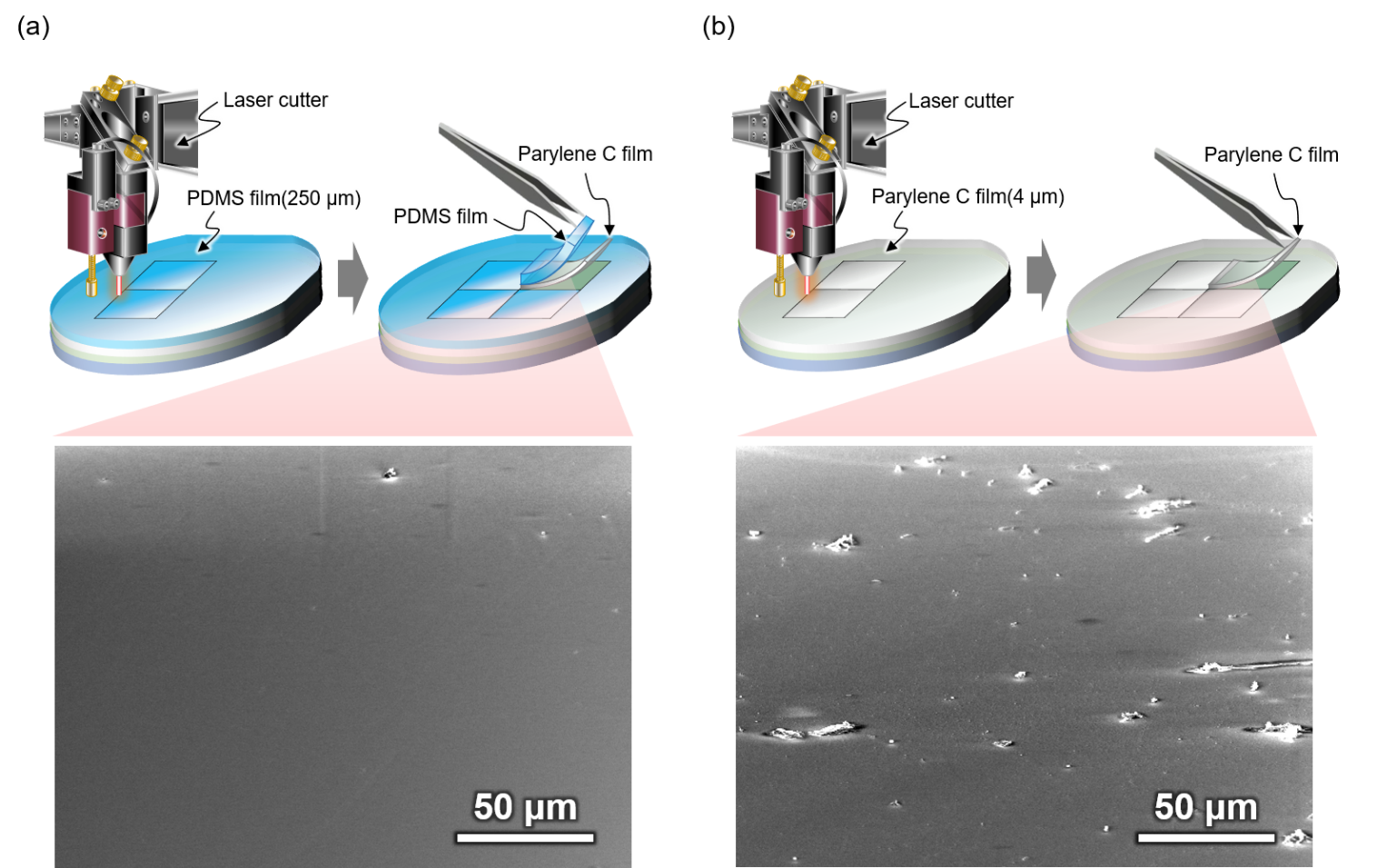


Figure S4. SEM images of the surface of parylene C film (a) with and (b) without PDMS film.


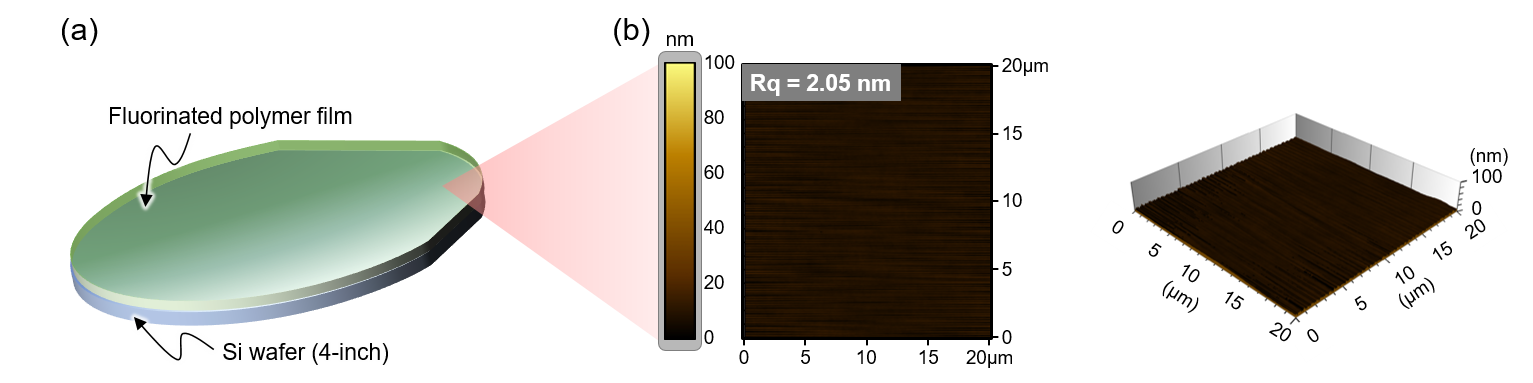


Figure S5. (a) Schematic diagram of silicon wafer surface coated with fluorinated polymer film and (b) roughness image of the surface using AFM.


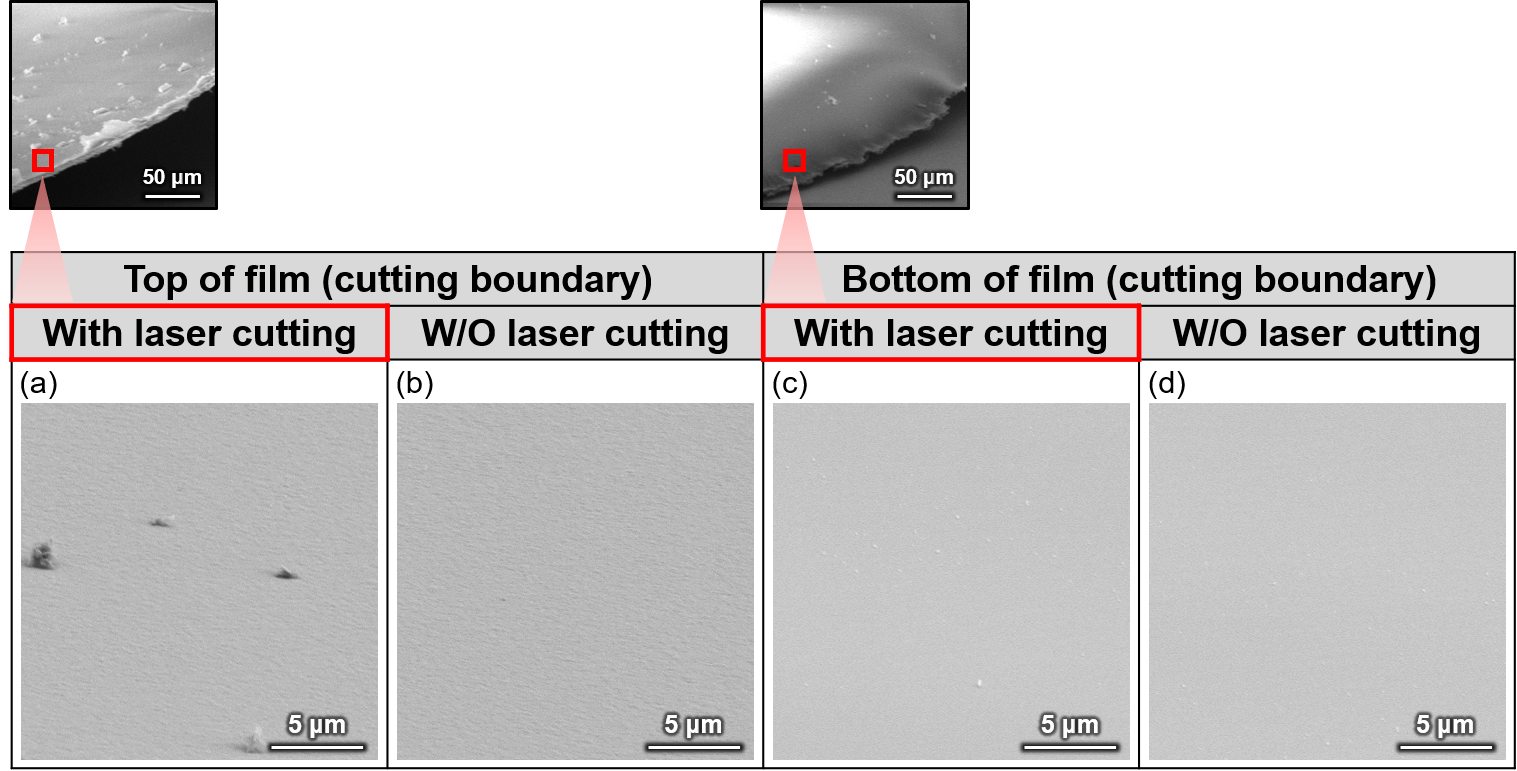


Figure S6. 10,000$\boldsymbol{\times}$ magnification SEM images of the cutting boundary surface at top and bottom of the film that went through the proposed laser patterning process (a), (c) and did not underwent the same (b), (d).

The SEM images in Fig. S6 were all observed at the cutting boundary. The laser cutting spec was applied in the same way as mentioned in Fig. S3. As a result, with and without laser cutting, hillocked morphology was confirmed on the top of the film, and in the case of laser cutting, some particles generated by ablation were attached to the surface as shown in Fig. S6a. Bubbles or small craters that may occur due to the thermal effect of the polymer were not found. Next, the bottom surface of the film in contact with the silicon wafer surface coated with fluorinated polymer film also had a relatively smooth surface in common with and without laser cutting. In conclusion, we have demonstrated that the laser cutting method presented in this article does not cause problems due to thermal effects on the roughness of the top and bottom surfaces of parylene C film except for some particles.

# References

1. Dean, J. A. *Lange’s Handbook Of Chemistry, 15th ed. McGraw-Hill: New York* (1999).
